# Zero-shot pseudowords memorability via representational content analysis

**DOI:** 10.3758/s13423-026-02875-x

**Published:** 2026-04-14

**Authors:** Daniele Gatti, Fritz Günther

**Affiliations:** 1 Department of Medicine and Surgery, University of Parma, Parma, Italy; 2 Institut für Psychologie, Humboldt-Universität zu Berlin, Berlin, Germany

**Keywords:** Memorability, Semantics, Distributional semantic models, Pseudowords

## Abstract

Novel strings of letters (i.e., pseudowords) lack established meaning(s), yet they may still evoke systematic, distributional signals that influence human behavior. Here, we tested whether distributional determinants of word memorability generalize to these novel strings. To do so, we leveraged a word-embedding model that was able to represent in a vector space not only attested words but also unmapped strings as bags of character *n*-grams. A ridge model trained on item-level word memorability norms learned a linear mapping from 300-dimensional embeddings to recognition memorability and achieved strong out-of-fold performance. We then applied this model zero-shot to predict memorability for 2,100 phonotactically legal pseudowords, whose baseline predictability was captured by orthographic and frequency features. Adding the zero-shot distributional score significantly improved the baseline model. These findings show that distributional representations derived from subword statistics carry mnemonic information that is not reducible to orthographic familiarity, and that novel strings are interpreted within a shared representational space learned from language experience. More broadly, they support the view that memorability is an intrinsic attribute predictable from representational information, even in the absence of learned meanings.

**Supplementary Information:**

The online version contains supplementary material available at 10.3758/s13423-026-02875-x.

## Introduction

Imagine that you are at a party, you meet a person called “Serena,” and you want to memorize their name. Many factors will influence your memory performance. Certain names are more *memorable* than others—for example, because they are infrequent and rare or because they are similar to existing words. Decades of research have traced back words’ memorability to items’ properties such as frequency or orthographic features (e.g., Brown et al., 1977; Madan, 2021).

Imagine now that you meet another person at the same party, but this person is called “Siro.”

This is the first time that you hear that name, and the processes underlying its memorability might not be as clear as for the other words that you know. These items (that can be called *novel words* or *pseudowords*) have no established meaning for a speaker that does not know them, and thus the factors driving their memorability are especially interesting. Recent work has shown that sublexical features such as length, orthographic neighborhood, and bigram and quadrigram frequency explain reliable variance in pseudowords memorability, suggesting that statistical familiarity and confusability are central determinants in this process (Woolnough & Tandon, 2025). This account broadly formalizes long-standing hypotheses about fluency and distinctiveness: strings composed of frequent letter sequences may be processed more efficiently, while those embedded in dense neighborhoods may be harder to individuate at test.

Moving away from orthographic features, recent studies have also shown, with a data-driven approach, the existence of distributional (semantic) determinants of words’ memorability (Aka et al., 2023). In their study, Aka and colleagues (2023) quantified words memorability by feeding a Ridge Linear Classifier with semantic features extracted by a language model. This technique has been recently labeled as “representational content analysis” (RCA) by Hussain and colleagues (2024), and it is an approach for interpreting the informational content of abstract numerical representations. Using this method, we aimed to investigate whether the same distributional determinants affecting words’ memorability can also generalize to pseudowords’ memorability.

The idea that although pseudowords have no explicit meaning they cannot be indicative of meaning recently has been challenged. That is, even though these stimuli have no place in the vocabulary, they can predictably activate semantic memory (e.g., Chuang et al., 2021; Gatti et al., 2023; Gatti, Raveling, et al., 2024a; Gatti, Rodio, et al., 2024b; Hendrix & Sun, 2021; Joosse et al., 2024). It is possible to quantify the semantic information triggered by these strings of letters through distributional semantic models (DSMs). DSMs represent word meanings as high-dimensional numerical vectors induced from large corpora of natural language under the assumption that the contexts in which words occur can approximate their meanings (Harris, 1954; Wittgenstein, 1953). Thus, words with similar meanings will be mapped to nearby points in a semantic space (Günther et al., 2019; Mandera et al., 2017). Pushing this forward, DSMs like *fastText* can be used to retrieve a representation for stimuli not proven in the training corpus by modelling them on the basis of the sequences of *n* contiguous letters (labeled as *n*-grams) composing it—that is, by quantifying the distributional patterns of their sublexical elements (Bojanowski et al., 2017). This approach has been used to approximate the “meaning” of pseudowords—that is, the semantic patterns that an unfamiliar letter string can elicit.

These premises suggest a simple hypothesis: If words’ and pseudowords’ semantics happen in a shared semantic space (e.g., Gatti et al., 2023), then a model trained to predict memorability from embeddings on real words might generalize to predict the memorability of novel pseudowords without task-specific fitting. Here, we test this directly. We trained a Ridge regression model on *fastText* embeddings of items drawn from established words’ memorability norms (Cortese et al., 2010, 2015) to predict item-level memorability for real words. We then apply the resulting model zero-shot to a set of pseudowords recently employed in a recognition memory study (Woolnough & Tandon, 2025). Because *fastText* composes vectors from character *n*-grams, every pseudoword receives a representation—even in the absence of a learned whole-word entry—allowing us to investigate whether subword-driven distributional cues alone can anticipate which pseudoword will be easier to recognize.

In this work, we focused specifically on the item-level distributional properties that affect recognition memory. We asked whether the way novel letter strings are embedded in a distributional space learned from prior language experience can account for systematic item-level differences in recognition performance, and thus the primary interest lay on these item-level differences. Our approach is therefore intended as a representational module that could be integrated into existing recognition memory frameworks (e.g., Reid et al., 2023), rather than as a full model of encoding, storage, retrieval, and decision.

Consistent with this, here memorability is treated as an item-level discriminability index derived from yes/no recognition performance. For each word or novel word, we used the hit rate minus the false-alarm rate, as reported in the source megastudies for words (Cortese et al., 2010, 2015) and in the recent novel words study (Woolnough & Tandon, 2025). Notably, these scores are computed by aggregating responses across many participants and lists, which yields an estimate of how easily each item is discriminated from foils on average, which is not affected by (possible) list- or individual-level effects. We therefore interpret memorability as an empirical item property that captures intrinsic variation in how consistently different word forms are remembered across individuals and settings.

## Experiment 1

### Material

Words’ memorability was obtained from the Cortese and colleagues’ databases (Cortese et al., 2010, 2015), which provide item-level recognition outcomes (i.e., hits and false alarms) for 5,578 monosyllabic and disyllabic English words collected from 234 participants. Memorability was operationalized as the delta between hits and false alarms.

In both the monosyllabic (2,578 items) and disyllabic (2,897 items) megastudies from Cortese and colleagues (2010, 2015), participants completed multiple study and test lists in a standard yes/no recognition memory paradigm. For example, in the disyllabic study, participants studied 30 lists of 50 words and were tested on 30 lists of 100 items (studied words intermixed with foils); item-level estimates of hits, false alarms, and hits minus false alarms (i.e., memorability) were then computed and used in multiple regression analyses.

### Distributional semantic model

The DSM used was *fastText* (Joulin et al., 2016; for a review, see Bonandrini & Gatti, 2024), and word vectors were retrieved from the English pretrained vectors (Bojanowski et al., 2017). The model was trained on English Wikipedia using the skip-gram method with 300 dimensions, character *n*-grams with a length of 3 to 6, and a window of size 5. *FastText* is an extension of the prominent *word2vec* model (Mikolov et al., 2013), a simple neural language model trained on a large corpus of natural language, with the training objective to predict the words immediately before and after a given word. To this end, *word2vec* uses a simple neural network model with one-hot vectors of the lexicon as input and output layers, and one hidden layer. After training, the weights between a given word in the input layer and the hidden layer (= the activation values of the hidden layer when exactly and only that word is present in the input layer) is taken as the word embedding (= vector representation) of that word. Since only the activation values of the hidden layer are directly used to predict the (context) words in the output layer, these activation values in the word embedding contain, in a condensed format, all the information required to make those predictions about (context) words. Thus, the word embedding encodes the distributional history of a word. Since words with similar distributions in language tend to have similar meanings, these vectors carry a lot of semantic information and are thus frequently employed as (distributed) semantic representations (Günther et al., 2019; Kumar, 2021).

With respect to traditional distributional models, whose ability to generate high-quality distributed semantic representations is limited to words that are sufficiently frequent in the input data, *fastText* is based on the idea (originally proposed by Schutze, 1992; and realized by Bojanowski et al., 2017, Fig. 1A) to take into account subword information by computing word vectors as the average of the semantic vectors for the *n-*grams associated with each word and the full word vector (when available). Crucially, this means that the word vectors can also be created for pseudowords, based on the subword units (i.e., *n*-grams) that they contain (Hendrix & Sun, 2021).

**Fig. 1:**
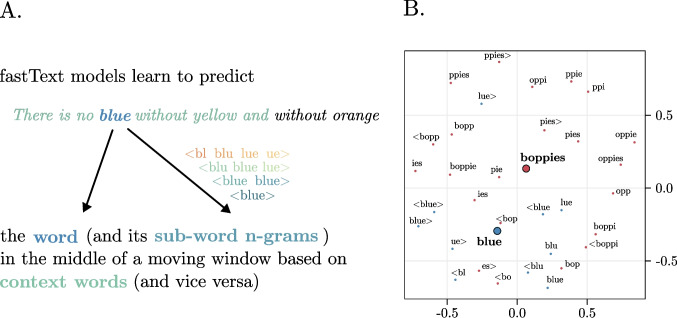
Graphical representation of how *fastText* computes word and sub-word vectors (**A**) and practical examples of how the vectors of the example word “blue” and novel word “boppies” (**B**) are computed as averaged vector (i.e., the centroid) of their embedded *n*-grams. (Color figure online)

As an example, consider the word <memory>, composed by different-length character *n*-grams, as reported in the Table 1. The *fastText*-induced representation will be the average of the <memory> word vector along with the vectors of the elements reported in Table 1 (depending on model’s characteristics, see also Fig. 1B).

**Table 1:** Example of the subword vectors that can be retrieved in order to represent the vector of the word <memory>. Note that the model employed here was bounded to 3-to-6-grams.

Word	Length (*n*)	Character *n*-grams
memory	1	m, e, m, o, r, y
memory	2	<m, me, em, mo, or, ry, y>
memory	3	<me, mem, emo, mor, ory, ry>
memory	4	<mem, memo, emor, mory, ory>
memory	5	<memo, memor, emory, mory>
memory	6	<memor, memory, emory>

The obtained vector dimensions capture the extent to which a target word is reliably predicted by the contexts in which it appears. Word vectors were retrieved using the *fastTextR* R package (Schwendinger & Hvitfeldt, 2022) and vector dimensions served as predictor set in subsequent analyses.

### Data analysis and results

To quantify semantic-based memorability, we used RCA, an approach for interpreting the informational content of abstract numerical representations (e.g., Hussain et al., 2024). Specifically, memorability was modeled from the 300-dimensions value associated to each word with Ridge regression, with $\alpha =0$ and 10-fold cross-validation. The cross-validated solution was summarized at *λ*_1se_ $-$ the largest penalty within 1 standard error of the minimum cross-validated error—trading a small increase in error for increased coefficient stability. For the selected $\lambda$, we took fold-out predictions ${\widehat{y}}_{i}$ and computed the following:

1 $$\mathrm{MSE}=\frac{1}{n}\sum\limits_{i=1}^{n}({y}_{i}-{\widehat{y}}_{i})^{2},{R}^{2}=1-\frac{\sum\limits_{i=1}^{n}({y}_{i}-{\widehat{y}}_{i})^{2}}{\sum\limits_{i=1}^{n}({y}_{i}-{\overline{y}})^{2}}$$

**Fig. 2:**
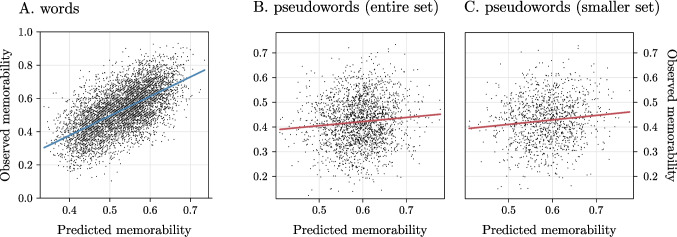
Results of Experiment 1 illustrating the relationship between observed word memorability and predicted one as learnt via RCA over distributional semantic information (**A**); results of Experiment 2 illustrating the relationship between observed pseudoword memorability and predicted one obtained from the model fitted on words (entire item set in **B**, true pseudowords only in **C**)

At *λ* =.12, performance was very good with *MSE* =.01, and *R*^2^ =.38 (Fig. 2A). This indicates that a linear mapping of *fastText* dimensions alone explains a substantial portion of item-level variance in word memorability under out-of-fold evaluation. Notably, via *fastText* embeddings we explain a larger portion of variance as compared with previous studies (i.e., Aka et al., 2023, had *R*^2^ =.25, although testing this only on a subset of items). From this fitted ridge model, we thus generated zero-shot memorability scores for the items included in Experiment 2.

We then evaluated the performance of this predicted memorability as learnt via RCA over distributional semantic information by adding it to a baseline model. The baseline model included as predictors i) number of characters (i.e., length), ii) mean orthographic distance to the 20 nearest real-word neighbors (OLD20 from the 20,000 most frequent words in the SUBTLEX-us), and iii) log-frequency as extracted from SUBTLEX-us (Brysbaert & New, 2009) and had *R*^2^ =.18. Pairwise correlations between the predicted memorability score and the baseline predictors were very weak or weak (i.e.,.04 < |*r*| <.36). Models were compared via Akaike information criterion (AIC^1^; Akaike, 1973). Notably, the full model outperformed the baseline model, ΔAIC = 2028.3, *F*(1,5568) = 2447, *p* <.001, and had *R*^*2*^ =.43 (see Supplementary Material for correlations among predictors). The effect of the predicted memorability score was significant, β =.55, *t* = 49.47, *p* <.001.

## Experiment 2

Experiment 2 evaluates whether the zero-shot distributional memorability scores trained on the Cortese database generalize to pseudowords memorability on the items included in Woolnough and Tandon (2025).

### Material

Memorability rates (again as hits − false alarms) for the 2,100 unique pseudowords included in Woolnough and Tandon (2025) were retrieved. In the original study, these pseudowords were all phonotactically legal and were retrieved from previous intracranial reading studies (Woolnough et al., 2021, 2022, 2023), from the English Lexicon Project (ELP; Balota et al., 2007) or from classic literature (e.g., Lewis Carroll, Roald Dahl, Dr. Seuss, A. A. Milne, Spike Milligan). A few real words (<100) of very low-frequency (meanings not widely known) were included in the original dataset. All stimuli were four to eight letters long and had SUBTLEX-US frequency <1 per million.

In Woolnough and Tandon (2025), participants completed a continuous recognition task in which 2,100 novel pseudowords were presented, some on multiple occasions, and participants indicated whether each was “old” or “new.” Consistent with Woolnough and Tandon (2025), we defined each novel word memorability as its hit-minus-false-alarm score, treated as an item-level discriminability index.

### Distributional semantic model

For consistency, pseudowords’ vectors were retrieved from the same DSM employed in Experiment 1. The zero-shot memorability score was then generated for the full set of unique items using the previously trained ridge model in Experiment 1. Among the starting 2,100 items, 860 were found to be attested in the English corpus used to train the DSM. That is, 860 items had a learned full vector in addition to subword ones. Given this, for a stricter analysis (see below), we focused on the subset of pseudowords that are completely unattested in the *fastText* model employed. Specifically, we distinguished between all pseudowords in the behavioral database, and out-of-vocabulary (OOV) pseudowords, defined as items that do not appear as lexical entries in the *fastText* training corpus (and thus have no pretrained word-level vector, only subword-based representations). Pseudowords that appear as tokens in the DSM training data (and therefore have direct word-level vectors) were excluded from the OOV subset but retained in the full-set analyses.

### Data analysis and results

In the original study by Woolnough and Tandon (2025), the authors predicted pseudowords memorability with a linear model including as predictors: i) number of characters (i.e., length), ii) mean orthographic distance to the 20 nearest real-word neighbors (OLD20), iii) highest and lowest corpus frequencies among the pseudoword’s closest real-word neighbors (hiFreqN/lowFreqN), and iv) mean two- and four-letter co-occurrence probabilities from SUBTLEX-US (bgf/qgf). This model had *R*^2^ =.074 and is used here as baseline.

**Fig. 3:**
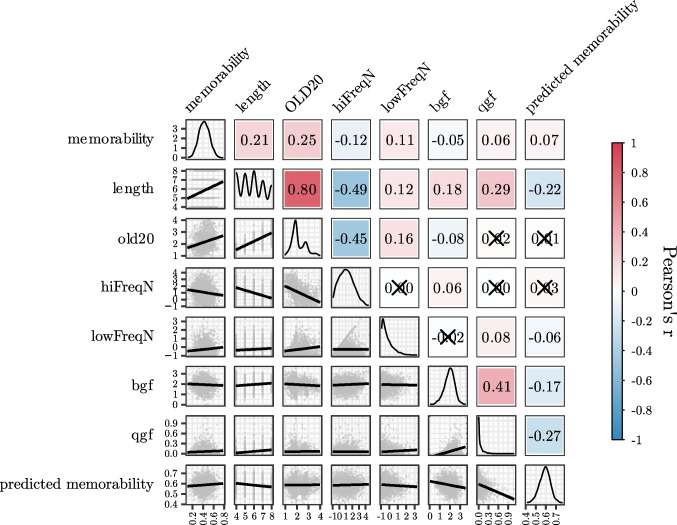
Correlation matrix among the dependent variable and the predictors considered in Experiment 2. Warmer colors indicate stronger positive correlation; crossed out cells indicate nonsignificant correlations. (Color figure online)

After retrieving baseline predictors from Woolnough and Tandon (2025) and fitting the baseline model, we then added to it the zero-shot memorability score obtained from pseudowords embeddings. Results showed that the model including the zero-shot memorability score outperformed the baseline one, ΔAIC = 15, *F*(1,2092) = 16.9, *p* <.001, and had *R*^2^ =.08. The effect of the zero-shot memorability score was significant, β =.09, *t* = 4.12, *p* <.001 (Fig. 2B). Notably, pairwise correlations between the zero-shot memorability score and the baseline predictors were very weak or weak (i.e.,.01 < |*r*| <.27; Fig. 3).

Then, this same procedure was repeated but including only OOV pseudowords. That is, by excluding the 860 items attested in the DSM employed. Results were consistent with those on the full dataset, with the baseline model having *R*^2^ =.12 and the model including the zero-shot memorability score having *R*^2^ =.13, thus outperforming it by ΔAIC = 11.2, *F*(1,1232) = 13.2, *p* <.001. Again, the effect of the zero-shot memorability score was significant, β =.11, *t* = 3.63, *p* <.001 (Fig. 2C). To assess whether the zero-shot memorability score was redundant with existing regressors, we examined multicollinearity using variance inflation factors (VIFs). The VIF for the zero-shot memorability was 1.19 in the former analysis and 1.24 in the latter, indicating extremely low collinearity with the other predictors. These results further show that our predictor captures information that is not redundant with the baseline, control variables, and, overall, these results indicate that semantic determinants of word memorability generalize to novel strings.

## Discussion

In the present study, we traced back pseudowords’ memorability to the distributional determinants affecting words’ memorability. Across two experiments, we first trained a Ridge model on existing words from established memorability norms which explained a substantial portion of variance. Then, we estimated a zero-shot memorability score for pseudowords that predicted item-level pseudoword memorability and explained unique variance beyond a strong orthographic baseline. Consistent with our expectations, these findings indicate that the distributional determinants affecting words’ memorability do generalize to pseudowords. Although the absolute improvement in explained variance from adding the representational predictor is modest (Δ*R*^2^ ~.01), this increment should also be interpreted relative to the (rather low) baseline explained variance for item-level pseudoword recognition (i.e., in our case a Δ*R*^2^ ~.01 corresponds to an ~10–15% relative increase in explained variance). Moreover, it should be noted that item-level recognition estimates for pseudowords are expected to be noisy (as compared with words) because the task involves discriminating highly confusable, unfamiliar strings; this constrains the attainable *R*^2^. Consistent with this, in Woolnough and Tandon (2025) the split-half reliability was *ρ* =.38.

These findings further extend previous evidence indicating that sublexical statistics predict pseudoword memorability (Woolnough & Tandon, 2025) by showing that a representation-learning approach—trained on real words—transfers to pseudowords with no task-specific fitting. Notably, the zero-shot generalization from words to pseudowords supports the idea that novel strings are interpreted within a shared representational space and that memorability can emerge from how an item projects into that space as shaped by experience, even when that item has never been encountered. Notably, prior work has shown that distributional vectors (can) encode information related to frequency, length, and other lexical variables (e.g., Hollis & Westbury, 2016; Westbury et al., 2025), and so if we are able to predict phenomena (such as memorability) from these vectors, it does not follow necessarily that this prediction is driven by semantic information. Critically, however, as our baseline model already includes length, frequency, and orthographical information, the remaining predictive gain from the zero-shot memorability index indicates that additional distributional information (such as semantic information) contributes to novel word memorability.

This effect speaks in favor of the idea that the information that pseudowords carry at the subword, distributional level can be activated implicitly even when there is no explicit request to manipulate it (for evidence of explicit manipulation, cf. Gatti, Raveling, et al., 2024a; Günther et al., 2026; Martínez-Tomás et al., 2025). Indeed, although pseudowords lack established lexical entries, there is converging evidence that semantic processing can be engaged implicitly by novel letter strings, suggesting that the system attempts semantic access even when lexical status is absent (e.g., Gatti et al., 2023; Hendrix & Sun, 2021). Overall, our results further support the idea that experience-derived form-meaning regularities influence the processing of novel, word-like stimuli. From this perspective, the present zero-shot distributional predictor may be interpreted as indexing how strongly a pseudoword taps into these learned (and implicit) distributional regularities, without necessarily assuming that participants explicitly derived meanings for the items.

Across domains, memorability appears to be an intrinsic attribute that is predictable from representational structure. In images, stable, cross-observer differences in what is remembered have been demonstrated and linked to interpretable features, indicating that memorability is not solely an epiphenomenon of transient attention (e.g., Isola et al., 2011). In verbal memory, classic fluency (frequency, sublexical familiarity) and distinctiveness (neighborhood sparsity, isolation) accounts explain complementary portions of variance (Alter & Oppenheimer, 2009; Hunt & Worthen, 2006) A growing literature adds semantic richness as a third determinant (Aka et al., 2023). Our results extend this to pseudowords: even absent learned meanings, subword-driven representational information as captured by *fastText* embeddings predicts which pseudowords will be remembered, consistent with the view that memorability reflects where stimuli land in a space learned from past experience.

In highlighting how distributional information affects novel words memorability beyond orthographic information, the present results fit naturally within global matching models of recognition memory, which assume that recognition strength depends on the global similarity between a probe’s representation and stored traces (e.g., Hintzman, 1984; Osth & Dennis, 2024; Osth et al., 2020). When these models are fed with orthographic or distributional representations, they can account for a range of word and novel word recognition phenomena. Reid and colleagues (2023), for example, showed that incorporating structured orthographic representations into a MINERVA2-style model explained item-based directed forgetting effects for novel words. Similarly, Osth and Zhang (2024) integrated word-orthographic representations with global similarity computation, combining orthographic and distributional information to explain item-level variability in recognition and false alarms for words. Here, we complement this previous work by demonstrating that within the domain of novel, non-lexical items the same type of distributional information affects memorability for known words. More broadly, the present findings extend the representational approaches above to the distributional properties of novel letter strings: by using subword-based embeddings to assign pseudowords positions in a vector space, we show that those positions predict item-level memorability above and beyond orthographic information.

In addition to MINERVA2-style global matching, our findings are also compatible with feature-copying recognition models such as retrieving effectively from memory (REM; e.g., Criss & McClelland, 2006; Shiffrin & Steyvers, 1997). In REM, each studied item is stored as an episodic trace composed of a vector of feature values, and encoding is assumed to be incomplete and error-prone (i.e., a noisy copy of the item’s features). At test, recognition is based on comparing the probe to all stored traces and evaluating evidence for an “old” versus “new” decision using a likelihood-based computation rather than a single undifferentiated strength value. Importantly for the present context, REM is largely agnostic about what counts as a “feature”: In principle, orthographic, sublexical, or embedding-derived distributional features can all be treated as inputs to the same matching-and-decision architecture, making it a natural theoretical home for combining orthographic and distributional representations in recognition memory.

In conclusion, our zero-shot memorability score derived from subword embeddings improves prediction of pseudoword memorability beyond a well-established orthographic baseline. That is, exposure-derived subword structure, as captured by *fastText*, carries mnemonic signals that are not fully reducible to orthographic neighborhood or *n*-gram frequency. This indicates that distributional representations—despite lacking learned meanings for novel strings—encode useful, generalizable regularities that align with human memory performance.

## Supplementary Information

Below is the link to the electronic supplementary material.

Supplementary file1 (DOCX 4247 KB)

## Data Availability

Data and codes used in the analysis are available at: https://osf.io/z43fy/overview?view_only=f7cac6befee440ee80e8c80d292f08c6 This study was not preregistered.

## Footnotes

1 This measure estimates the quality of a model and balances the trade-off between the goodness of fit of a model and its complexity, aiming to select a model that both explains the data well and is not overly complex. The AIC can be used to compare different models fitted on the same set of data and its direction is informative of the best model in the pool of the estimated ones (i.e., the model with the lowest the AIC is the best one).

## Declarations

### Prior dissemination

A previous version of this article was uploaded on PsyArXiv (https://osf.io/preprints/psyarxiv/cvxgf_v1).
