# Supplementary material for: Zero-shot pseudowords memorability via representational content analysis
Source: Psychon Bull Rev. 2026 Apr 14;33(4):137. doi: 10.3758/s13423-026-02875-x (PMC13079521; doi:10.3758/s13423-026-02875-x)
Supplement: Supplementary file 1 — Supplementary file1 (DOCX 4247 KB) [file 13423_2026_2875_MOESM1_ESM.docx]

**Supplementary Material**

**Figure SM1.** Correlation matrices of the dependent variable and the predictors considered in Experiment 1 (A, B).
